# Risk factors associated with neonatal deaths: a matched case–control study in Indonesia

**DOI:** 10.3402/gha.v9.30445

**Published:** 2016-02-16

**Authors:** Asnawi Abdullah, Krishna Hort, Yuli Butu, Louise Simpson

**Affiliations:** 1 Faculty of Public Health, University Muhammadiyah Aceh, Banda Aceh, Indonesia; 2 Australia Indonesia Partnership for Maternal and Neonatal Health, Kupang, Indonesia; 3 Department of Epidemiology and Preventive Medicine, Monash University, Clayton, Australia; 4 Nossal Institute for Global Health, University of Melbourne, Parkville, Australia

**Keywords:** neonatal death, risk factors, matched case–control study, maternal health and neonatal health, Indonesia

## Abstract

**Background:**

Similar to global trends, neonatal mortality has fallen only slightly in Indonesia over the period 1990–2010, with a high proportion of deaths in the first week of life.

**Objective:**

This study aimed to identify risk factors associated with neonatal deaths of low and normal birthweight infants that were amenable to health service intervention at a community level in a relatively poor province of Indonesia.

**Design:**

A matched case–control study of neonatal deaths reported from selected community health centres (*puskesmas*) was conducted over 10 months in 2013. Cases were singleton births, born by vaginal delivery, at home or in a health facility, matched with two controls satisfying the same criteria. Potential variables related to maternal and neonatal risk factors were collected from *puskesmas* medical records and through home visit interviews. A conditional logistic regression was performed to calculate odds ratios using the clogit procedure in Stata 11.

**Results:**

Combining all significant variables related to maternal, neonatal, and delivery factors into a single multivariate model, six factors were found to be significantly associated with a higher risk of neonatal death. The factors identified were as follows: neonatal complications during birth; mother noting a health problem during the first 28 days; maternal lack of knowledge of danger signs for neonates; low Apgar score; delivery at home; and history of complications during pregnancy. Three risk factors (neonatal complication at delivery; neonatal health problem noted by mother; and low Apgar score) were significantly associated with early neonatal death at age 0–7 days. For normal birthweight neonates, three factors (complications during delivery; lack of early initiation of breastfeeding; and lack of maternal knowledge of neonatal danger signs) were found to be associated with a higher risk of neonatal death.

**Conclusion:**

The study identified a number of factors amenable to health service intervention associated with neonatal deaths in normal and low birthweight infants. These factors include maternal knowledge of danger signs, response to health problems noted by parents in the first month, early initiation of breastfeeding, and delivery at home. Addressing these factors could reduce neonatal deaths in low resource settings.

## Background

The neonatal mortality rate in developing countries continues to be an urgent global problem with over 4 million infants dying within the first month of life (1). Indonesia is part of this global trend, with neonatal mortality rates (NMRs) falling by only 3% annually between 1990 and 2012 (2). The latest Indonesian Demographic and Health (IDHS) survey, conducted in 2012, estimated an NMR of 19/1,000 live births for the 5-year period preceding the survey, a rate that is unchanged from the IDHS survey of 2007 and only slightly lower than the estimate of 20/1,000 from the 2002–2003 survey (3). The estimated NMR in Indonesia of 14/1,000 in 2014 is still higher than in some other Southeast Asian countries; it is twice that of Thailand (7/1,000) and three times higher than Malaysia (4/1,000) (2). There is considerable regional variation in neonatal deaths (4), as well as between different socio-economic groups (5, 6) within Indonesia. The 2012 IDHS showed the NMR in some provinces in eastern Indonesia to be three times higher than in western Indonesia (3).

Nusa Tenggara Timur (NTT) is one of the provinces in eastern Indonesia with a high rate of neonatal death (26/1,000 live births) compared to the national rate of 20/1,000 live births (3). Several innovative programmes have been introduced to improve maternal and neonatal health in NTT. In addition to national maternal and child health programmes (7), the *Revolusi KIA* (Maternal Neonatal Child Health Revolution) initiative was launched in 2009 (8) by the NTT government. In the same year, the Australia–Indonesia Partnership for Maternal and Neonatal Health (AIPMNH) project commenced (9). By working collaboratively with government and other partners, major improvements to the maternal and neonatal health systems have been achieved, particularly in the 14 AIPMNH-assisted districts. These improvements include expansion of basic obstetric and neonatal emergency care to 72 of 286 community health centres (*puskesmas*) and expansion of comprehensive obstetric and neonatal emergency care at 11 district hospitals.

Although the number of reported maternal deaths in NTT fell by 40% between 2009 and 2014, the number of neonatal deaths fell by only 8% over the same period. Because Indonesia does not yet have complete recording of maternal or neonatal deaths through a vital registration system, the number of reported deaths reflects those reported to the health services and is likely to underestimate population death rates. Comparison with population estimates from surveys such as the IDHS indicates that the reported neonatal death rate of 10/1,000 for the period 2012–2014 was less than 50% of the IDHS survey estimate of 26/1,000 for the period 2007–2012. Of neonatal deaths reported in NTT in 2013, approximately 65% were deaths reported from hospitals, although, on average, only about 20% of estimated births occur in district hospitals. Although reporting from the hospitals provides information on causes and factors related to hospital neonatal deaths, there is little information on causes and factors responsible for neonatal deaths occurring outside the hospitals.

With AIPMNH support, the NTT district health offices introduced a collection of basic data on maternal and neonatal deaths reported through an audit process. A review of 2,246 cases of neonatal deaths reported from the 14 AIPMNH districts between 2012 and 2014 found an increasing proportion of deaths in normal birthweight neonates (from 42% of deaths in 2012 to 49% in 2014). Most deaths occurred in the first 7 days of life (84% in 2012, 79% in 2013, and 82% in 2014), although this still left between 16 and 21% occurring after Day 7. Averaging these reported deaths over the 3 years, 44% delivered in hospital, 36% in a community health facility, and 18% at home, whereas 65% died in hospital, 12% in a health facility, and 22% at home.

Current information on risk factors associated with neonatal deaths in Indonesia are mainly derived from analysis of national surveys (4, 5, 10). These analyses identified that the factors associated with neonatal deaths are low birthweight, low household income, high birth order, and complications during delivery (haemorrhage, eclampsia, and infection). These findings focus attention on the management of high-risk pregnancies and complications of delivery. However, given the data obtained through the audit process on the management of newborns who had died, we were concerned with identifying factors in the management of these newborns that could be addressed by community-level health service interventions. Our focus on the reported deaths was based on the assumption that these deaths occurred in families who had at least some contact with health services and thus the potential to be reached by health service interventions. We were particularly interested to identify interventions that could address deaths in low birthweight babies and those surviving beyond 7 days.

The aim of this study was to identify risk factors for neonatal deaths reported to health services that were potentially amenable to health service intervention by community-level services. Of particular interest was to explore whether risk factors might differ between normal and low birthweight infants and between infants dying in the first week compared to those surviving beyond 7 days.

## Methods

We selected a study design that would enable us to compare the frequency of known risk factors between neonates who survived and those who died, while controlling for other known risk factors outside direct health service intervention, such as low birthweight and socio-economic disadvantage. For these reasons, we selected a case–control study design. We used the list of reported neonatal deaths for selection of cases and selected controls from the same village (and thus similar socio-economic conditions and access to services) and with the same birthweight category (normal or low birthweight) as those who died. We also focused on the lower risk group of neonates by excluding multiple births and births resulting from caesarean section.

### Study design

Cases were defined as neonatal deaths reported to health services during the period between 1 January to 30 October 2013 that were singletons and non-caesarean-section delivery. Cases were selected from the list of neonatal deaths reported as part of the neonatal audit, from the 80 *puskesmas* across the 14 districts that were directly supported by the AIPMNH programme. In each *puskesmas*, cases that satisfied the criteria were included where the mother was alive, could be located, and was willing to be interviewed.

Each case was matched with two controls born in the same period of the month, from the same village and the same birthweight category, 1) normal weight and 2) low birthweight (<2,500 grams). Matching was done using the cohort register kept at the *puskesmas* and identifying the next suitable birth that resulted in a surviving infant from the same village and with the same birthweight category.

The neonatal mortality records from the 14 districts identified 240 neonatal deaths reported during the study period. However, only 169 cases could be identified for follow-up during the study, as the remaining cases could not be located at their recorded address. In addition, in 15 cases only one suitable control could be identified, and these were also removed from the analysis, leaving 154 cases and 308 controls. Calculations of the ability of this sample size to detect differences between cases and controls identified that 154 cases and 308 controls would be sufficient to detect a minimum odds ratio of 2.0 for risk factors occurring in 25% of controls, with a probability of 5% and power of 90%.

### Data collection and questionnaire

From the audit data on neonatal deaths, 154 cases were selected based on the criteria for inclusion and 308 matched controls (surviving neonates) identified from corresponding *puskesmas* records. For both cases and controls, primary data from *puskesmas* records was collected, followed by home-based interviews with mothers.

Primary data included the following: mother's age, previous pregnancies; baby's birthweight; place of birth; assistant at birth; Apgar score at birth; record of antenatal care; record of complications in previous pregnancies; complications during pregnancy; maternal complications at birth; neonatal complications at birth; early initiation of breastfeeding; and use of kangaroo method of care.

Interview data included the following: socio-economic indicators; knowledge of high-risk pregnancy; danger signs in newborns; use of a traditional birth attendant (TBA); practices during pregnancy; care of umbilical cord; and post-partum care.

Interviews were conducted by *puskesmas* midwives using a questionnaire that focused on factors not available from routine *puskesmas* data. As part of study preparation the questionnaire was pretested and piloted in a *puskesmas* and a village, and once finalised specific training in use of the instrument was provided to midwives conducting the data collection.

### The theoretical framework and variables

The theoretical framework was adopted from previous studies conducted in developing countries (4, 11–15) and was the basis for determining potential risk factor variables. Theoretically, neonatal death may be associated with maternal risk factors, neonatal risk factors, health system factors, and socio-economic determinants. A neonatal death is defined as any death occurring during the first 28 completed days of life, with early neonatal deaths being those occurring in the first 7 days and late neonatal deaths those 8 to 28 days after birth. Predictor variables were those related to maternal characteristics, maternal knowledge, maternal health, neonatal characteristics, delivery factors, and variables outside the control of health services. These variables are described in detail in the following sections and in Table 1.

**Table 1:** Data sources and grouping for analysis

Factor grouping	Data (analytic categories)		Data source
Maternal factors	Maternal knowledge	Use of iron tablets during pregnancy (completed 90 days: yes/no)	Interview with mother
		Knew whether pregnancy was high risk (able to name high risk conditions: yes/no)	
		Knew danger signs of pregnancy and delivery (able to name one sign such as bleeding, convulsions, fever, etc.: yes/no)	
		Informed of estimated date of delivery (informed/not informed)	
		Knew danger signs of newborn (able to name one sign such as fever, convulsions, diarrhoea, difficulty breathing: yes/no)	
	Maternal health during pregnancy	Any illness during pregnancy? (yes/no)	Interview with mother
		Any complications during pregnancy? (e.g. bleeding, pre-eclampsia, CPD: yes/no)	Antenatal record
		Any complications during delivery? (yes/no)	Health centre/delivery record
	Maternal characteristics	Age at marriage, age at pregnancy	ANC record
		History of previous pregnancies, including previous abortions	ANC record+interview
		Haemoglobin during pregnancy	ANC record
		Risk status during pregnancy	ANC record+interview
Delivery	Location	Home/healthcare facility	Record+interview
	Assistance at delivery	Assisted by TBA/nurse/midwife or doctor	Record+interview
Neonatal	Characteristics	Gender, birthweight	Record
	Complications	Complications at delivery	Record
	Health problem	Problem requiring visit to healthcare provider	Interview
	Apgar score	Where available	Record
	Practices	Early initiation breastfeeding, kangaroo method of care	Record
Variables outside health service	Geography	Distance and time of travel from village to *puskesmas* (>20 km/<20 km, >60 minutes travel/<60 minutes)	Interview with mother
	Education	Highest educational level of mother and father (graduated primary school: yes/no)	Interview with mother
	Economic status	Condition of house, access to electricity, monthly income	Interview with mother

CPD, cephalopelvic disproportion; ANC, antenatal care.

#### Maternal factors

Maternal factors were grouped into three aspects: maternal knowledge (mother's knowledge of risk and danger signs in pregnancy, childbirth, and newborns); maternal health (mother's report of illness during pregnancy and complications noted in medical records); and maternal characteristics (age, previous pregnancies, and antenatal care).

#### Neonatal factors

Neonatal factors included gender, record of complications during birth delivery, APGAR score, mother's report of health problems after birth, early initiation of breastfeeding, and use of the kangaroo method of care.

#### Delivery factors

Delivery factors consisted of place of birth delivery (at home or in a healthcare facility), and assistance during birth delivery.

#### Variables outside control of health services

Variables outside the control of health services were distance to *puskesmas*, time to *puskesmas*, highest education level of parents, and poverty indicators.

### Statistical analysis

A conditional logistic regression model (16, 17) was used to calculate the crude odds ratio (COR) and adjusted odds ratios (AOR) with 95% confidence intervals (CIs) for any association with neonatal death. The analysis was conducted using the clogit procedure in STATA 11 (18). The analysis began with univariate analysis to identify the variables that reached a statistically significant association with neonatal death. The variables found to be significant at *P*≤0.20 were included in a multivariate analysis. A stepwise (with *P=*0.20) approach was performed to select variables for inclusion in modelling. Multivariate analysis was conducted for each individual factor (i.e. maternal characteristics, maternal knowledge, maternal health, and neonatal factors). Four multivariate models were generated, with the first model including variables related to place of birth delivery and maternal knowledge. The second model included variables in the first model plus variables related to maternal health during pregnancy. The third model included all maternal risk factors (knowledge, maternal health during pregnancy, and maternal characteristics). The fourth and final model included all the variables in the previous models plus neonatal factors. All models were adjusted for the variables outside control of health services found to have a significant relationship with neonatal death on univariate analysis at *P*=0.02 (time to community health centre, parents’ highest education level, and electricity ownership).

### Sub-analysis of early neonatal death

In addition to the main analysis, two subgroup analyses were performed: 1) sub-analysis of early neonatal death (within 7 days of birth) and late neonatal death (8–28 days after birth); 2) sub-analysis of normal and low birthweight (<2,500 g) neonates. All variables reaching a significance level of *P*=0.20 in the multivariate analysis in the main model were included in the subgroup analyses.

### Missing data

The variables of monthly household income, birth spacing, haemoglobin, history of having a neonatal complication, Apgar score, and use of the kangaroo method of care had >10% missing values and these were imputed by creation of a missing value (99), so that all cases and controls were included in the analyses.

### Sensitivity analysis and assessment of model

A sensitivity analysis was conducted by including and excluding the individuals with missing information. Interaction between variables was investigated and any significant interaction was included in the model (i.e. complication during pregnancy and neonatal complication at birth). The potential for interactions was examined only when the suspected variables were retained in the multivariate model.

### Ethics statement

The protocol was approved by the local government ethics committee at the University of Cendana, NTT. Informed consent was obtained from all respondents prior to the interview.

## Results

### Characteristics of the sample

A total of 154 cases of neonatal death and 308 surviving neonates were included in the main analysis of this study. The average birthweight was 2,591 grams (range 1,000–4,900 grams), 204 (44%) were low birthweight (<2,500 grams), and 74% of neonatal deaths occurred in the first week of life. Cases and controls were selected from 220 villages across 70 *puskesmas* in the 14 AIPMNH-assisted districts. The average respondent household income per month was IDR 734,248 (USD 60). Approximately 16% of mothers and 10% of fathers had not graduated from primary school. The median distance of respondents to a *puskesmas* was 3 kilometres with a median access time of approximately 20 minutes. Almost 50% of respondents had no access to their own electricity (own meter box). Among several key socio-economic variables, the availability of electricity (as a proxy poverty factor) was found to be independently associated with a higher risk for neonatal death. Other variables are listed in Table 2.

**Table 2:** Proportion and univariate conditional logistic regression analysis of factors associated with neonatal death (0–28 days)

	Neonatal death 0–28 days			
	
Factors and variables	Case *n* (%)	Control *n* (%)	Odds ratio (95% CI)	*P*
**1. Delivery factors**				
Place of delivery				
In healthcare facility	117 (76)	291 (94)		
At home	35 (23)	17 (6)	5.26 (2.72–10.20)	0.000
Birth delivery provider				
Healthcare provider	123 (81)	293 (95)		
Traditional birth attendant	29 (19)	14 (5)	4.90 (2.44–9.86)	0.000
**2. Maternal knowledge**				
Taking all recommended iron supplements				
Yes	129 (84)	280 (91)		
No	23 (15)	26 (8)	2.27 (1.13–4.54)	0.021
Knowledge of high risk pregnancy				
Yes	100 (65)	222 (73)		
No	53 (35)	82 (27)	1.59 (0.96–2.64)	0.069
Knowledge of danger signs during pregnancy				
Yes	117 (77)	255 (83)		
No	35 (23)	52 (17)	1.51 (0.89–2.55)	0.123
Knowledge of due date				
Yes	143 (93)	300 (97)		
No	11 (7)	8 (3)	2.96 (1.14–7.69)	0.026
Knowledge of danger signs in newborns				
Yes	84 (55)	204 (66)		
No	70 (45)	104 (34)	1.88 (1.19–2.97)	0.007
**3. Maternal health**				
Complications during pregnancy?				
No	134 (87)	293 (95)		
Yes	20 (13)	15 (5)	2.88 (1.42–5.82)	0.003
Complications at birth?				
No	120 (78)	282 (92)		
Yes	34 (22)	26 (8)	3.38 (1.86–6.13)	0.000
Illness during pregnancy?				
Never	101 (66)	224 (73)		
Ever	53 (34)	84 (27)	1.51 (0.95–2.40)	0.083
Malaria				
No	136 (88)	280 (91)		
Yes	18 (12)	28 (9)	1.4 (0.7–2.9)	0.327
**4. Maternal characteristics**				
Age at birth delivery				
20–35	110 (71)	231 (75)		
<20	11 (7)	30 (10)	0.77 (0.37–1.60)	0.487
>35	33 (21)	47 (15)	1.50 (0.90–2.50)	0.123
Birth spacing				
More than 2 years	75 (49)	146 (47)		
Less than 2 years	20 (13)	31 (10)	1.27 (0.68–2.38)	0.453
Age at marriage (first)				
Over 20	132 (86)	288 (94)		
20 or under	20 (13)	19 (6)	2.41 (1.21–4.79)	0.012
Previous abortion?				
No	135 (88)	287 (93)		
Yes	19 (12)	21 (7)	1.91 (0.99–3.68)	0.052
Gravida				
Five or less	129 (88)	280 (95)		
More than five	17 (12)	15 (5)	2.43 (1.20–4.93)	0.014
High risk pregnancy?				
No	92 (61)	229 (76)		
Yes	59 (39)	71 (24)	2.01 (1.32–3.07)	0.001
Haemoglobin				
Normal	29 (19)	77 (25)		
Low haemoglobin (<11)	61 (40)	121 (39)	1.57 (0.85–2.91)	0.153
**5. Neonatal factors**				
Sex				
Female	57 (38)	151 (50)		
Male	93 (62)	153 (50)	1.60 (1.07–2.39)	0.022
Neonatal complications during birth delivery?				
No	49 (32)	265 (86)		
Yes	105 (68)	43 (14)	27.02 (11.81–61.82)	0.000
History of neonatal complications?				
No	80 (52)	173 (56)		
Yes	24 (16)	18 (6)	2.94 (1.48–5.82)	0.002
Missing	50 (32)	117 (38)	0.88 (0.56–1.39)	0.590
Apgar score				
Normal	49 (32)	263 (85)		
Low (<7)	61 (40)	24 (8)	16.49 (7.80–34.85)	0.000
Missing	44 (29)	21 (7)	16.62 (7.12–38.82)	0.000
Had health problems and visited healthcare provider?				
No	54 (35)	248 (81)		
Yes	74 (48)	54 (18)	6.64 (3.90–11.30)	0.000
Missing	26 (17)	6 (2)	18.39 (6.97–48.48)	0.000
Initiated early breastfeeding?				
Yes	48 (31)	244 (79)		
No	106 (69)	64 (21)	10.46 (5.93–18.45)	0.000
Practiced kangaroo method?				
Yes	24 (16)	74 (24)		
No	105 (68)	181 (59)	2.31 (1.24–4.32)	0.009
No relevant response or missing	25 (16)	53 (17)	1.74 (0.81–3.71)	0.153
**6. Variables outside control of health services**				
Accessibility				
Distance to community health centre				
<20 km	129 (87)	268 (92)		
≥20 km	20 (13)	24 (8)	2.79 (1.00–7.72)	0.049
Time to community health centre				
Less than 1 hour	116 (75)	250 (81)		
More than 1 hour	38 (25)	58 (19)	2.37 (1.09–5.17)	0.030
Education				
Mother graduated from primary school?				
Yes	125 (81)	264 (86)		
No	29 (19)	44 (14)	1.44 (0.83–2.47)	0.192
Both mother and father graduated from primary school?				
Yes	115 (86)	285 (93)		
No	19 (14)	23 (7)	1.86 (0.94–3.69)	0.077
Poverty variables				
Electricity ownership				
Yes	66 (43)	164 (53)		
No	88 (57)	144 (47)	1.76 (1.11–2.79)	0.016
Poverty indicator (dirt floor and no electricity)				
Own electricity and no dirt floor	110 (71)	242 (79)		
Dirt floor and no electricity	44 (29)	66 (21)	1.56 (0.96–2.52)	0.072
Income per month				
Over 1 million IDR	17 (11)	46 (15)		
1 million IDR or less	109 (71)	213 (69)	1.48 (0.76–2.91)	0.249
Social support				
Living alone?				
Yes	101 (66)	174 (56)		
No (living with family)	51 (33)	131 (43)	0.64 (0.41–0.99)	0.043

CI, confidence interval; IDR, Indonesian rupiah.

### Risk factors for neonatal death

The variables found to be associated with a higher risk for neonatal death are summarized in Table 2 (bivariate) and Table 3 (multivariate). These variable include the following: (a) four variables related to maternal factors: inadequate maternal knowledge of neonatal danger signs; complications at the time of delivery; age at marriage; and a history of abortion; (b) three variables related to neonatal factors: neonatal complications during delivery; neonates having a health problem during the first 28 days; and a low Apgar score; (c) one variable related to delivery factors identified as significant was delivery at home. Combining all significant variables related to delivery, maternal, and neonatal factors into a single multivariate model, six risk factors were found to be significantly associated with a higher probability of neonatal death. The risk factors identified were the following: neonatal complication during birth; having a health problem during the first 28 days; maternal lack of knowledge of danger signs for neonates; low Apgar score; delivery at home; and complications during pregnancy.

**Table 3:** Multivariate conditional logistic regression analysis of maternal factors associated with higher risk of neonatal death at 0–28 days^a^

Variables	Model 1	Model 2	Model 3	Model 4
Place of delivery				
Home birth vs. at healthcare facility	4.30 (2.12–8.74)***	4.27 (2.08–8.76)***	5.05 (2.26–11.25)***	8.65 (1.11–67.49)*
Maternal knowledge				
Lack of knowledge about/did not take iron supplements	1.52 (0.70–3.26)	1.41 (0.62–3.18)	1.43 (0.60–3.43)	1.30 (0.20–8.55)
No or limited knowledge about due date	2.08 (0.70–6.14)	1.45 (0.46–4.50)	1.58 (0.43–5.85)	0.92 (0.09–9.44)
Lack of knowledge of danger signs for newborns	1.40 (0.85–2.32)	1.51 (0.89–2.56)	1.32 (0.76–2.31)	5.90 (1.32–26.47)**
Maternal health				
Complications during pregnancy		2.09 (0.95–4.61)	1.77 (0.78–4.03)	2.84 (0.39–20.68)
Complications during birth		3.72 (1.92–7.23)***	3.52 (1.74–7.08)***	1.41 (0.17–11.69)
Maternal characteristics				
Age at (first) marriage			2.57 (1.07–6.21)*	2.20 (0.13–35.77)
Previous abortion			2.00 (0.91–4.41)	0.82 (0.09–7.42)
High risk pregnancy			1.59 (0.94–2.70)	1.86 (0.54–6.36)
Neonatal factors				
Complications during birth				41.20 (5.12–331.29)***
History of mother having neonatal complications				5.91 (0.81–42.94)
Low Apgar score				7.48 (1.31–42.67)*
Health problems				15.07 (3.28–69.19)***
No early breastfeeding initiation				1.41 (0.39–5.03)
Factors outside control of health system				
Accessibility: time to community health centre of more than 1 hour	1.67 (0.73–3.82)	2.28 (0.93–5.62)	2.53 (0.99–6.49)	3.73 (0.49–28.60)
Education: both wife and husband did not graduate primary school	1.32 (0.61–2.84)	1.18 (0.54–2.59)	0.86 (0.37–2.02)	1.26 (0.19–8.52)
Poverty indicator: parents do not have their own electricity meter	1.63 (0.98–2.72)	1.66 (0.97–2.85)	1.77 (1.00–3.13)	2.83 (0.75–10.60)

a Pseudo *R*^2^=81.3%;

* significant at *P*<0.05;

** significant at *P*<0.001;

*** significant at *P*<0.0001. Bayesian information criterion for Models 1–4: 347.15, 337.85, 338.53, and 201.283, respectively. Akaike information criterion for Models 1–4: 309.93, 292.36, 277.02, and 106.97, respectively.

### Sub-analysis

In our study, 74% of neonatal deaths occurred in the first week of life (early neonatal death). In the first subgroup analysis of early neonatal death category (*n*=345), the main risk factors were neonatal complications during birth, low Apgar score, and the neonate having a health problem. All were found to be statistically significantly associated with higher risk for early neonatal death. For the group of neonates who died between 7 and 28 days after birth (late neonatal death) (*n*=117), the following risk factors were significantly associated with a higher risk of death: lack of maternal knowledge of neonatal danger signs; previous history of complications; and lack of early initiation of breastfeeding.

In the second subgroup analysis, among the group of normal birthweight neonates (*n*=258), three variables were significantly associated with a higher risk of neonatal death: complications during delivery; lack of early initiation of breastfeeding; and lack of maternal knowledge of neonatal danger signs. For neonates in the low birthweight category (*n*=204), six variables were found to be significantly associated with neonatal death: neonatal complications at delivery; the newborn having health problems; delivery at home; lack of maternal knowledge of neonatal danger signs; not using the kangaroo method of care; and low Apgar score. The comparison of risk factors for different subgroup analysis is presented in Table 4.

**Table 4:** Variables associated with a higher risk of neonatal death in multivariate subgroup analysis adjusted for socio-economic status

Risk factors	Early neonatal death (*n*=345)	Late neonatal death (*n*=117)	Low birthweight (*n*=204)	Normal birthweight (*n*=256)
Neonatal complication at birth	18.8 (4.2–84.5)***	123.2 (3.8–3,974)**	262.8 (4.5–15,317)**	194.4 (7.81–4,842)**
Neonate having health problem	4.2 (1.3–13.6)*		195.5 (2.7–14,306)*	7.8 (0.99–61.7)
Lack of knowledge of danger signs for newborns		8.1 (1.2–57.9)*	37.7 (2.1–686.8)*	13.4 (1.1–164.8)*
Neonate having low Apgar score	4.9 (1.2–20.5)**		27.9 (1.3–618)*	
Maternal complications during pregnancy			6.3 (0.4–99.0)	57.5 (0.6–5,418)
Delivery at home	4.4 (0.9–20.7)		171.2 (1.8–16,296)*	
History of maternal having neonatal complications		78.4 (2.0–3,046)*	7.9 (0.6–101.6)	
Neonate did not receive kangaroo method			32.1 (1.1–952)*	
Neonate did not receive early breastfeeding		44.2 (2.1–933)*		20.9 (2.5–173.7)**
Maternal high risk pregnancy			5.9 (0.6–54.1)	
Age at marriage (first)	6.5 (0.6–63.5)			

* Significant at *P*<0.05;

** significant at *P*<0.001;

*** significant at *P*<0.0001.

*Note:* Only variables that were significant at *P*<0.20 were included.

Sensitivity analysis found that inclusion or exclusion of missing values for variables with a high proportion of missing values (haemoglobin, Apgar) had no significant effect on the overall results. A series of collinearity test was conducted and the results (Tolerance/Condition Number/Determinant Correlation Matrix) showed no overlapping or collinearity. Tests for goodness of fit of Bayesian information criterion and Akaike information criterion demonstrated that the final model (Model 4) had satisfactory scores (the lowest score).

## Discussion

### Key findings of risk factors

This study found 11 main risk factors statistically significantly associated with neonatal death. The risk factors were as follows: 1) neonatal complications at delivery (as noted on medical records); 2) neonatal health problem requiring a visit to a healthcare provider (as reported by mother); 3) lack of maternal knowledge of neonatal danger signs; 4) low Apgar score; 5) maternal complications during pregnancy (as noted on medical records); 6) delivery at home; 7) history of complications in previous pregnancies (as noted on medical records); 8) not using the kangaroo method of care; 9) not receiving early initiation of breastfeeding; 10) a high-risk maternal pregnancy; and 11) mother's age at marriage. Six risk factors (1, 2, 3, 4, 5, and 6) were significantly associated with a higher risk of neonatal death at 0–28 days. Three risk factors (1, 2, and 4) were significantly associated with early neonatal death (0–7 days). Four risk factors (1, 3, 7, and 9) were significantly associated with late neonatal death.

For neonates born with low birthweight, six risk factors (1, 2, 3, 4, 6, and 8) were found to be significantly associated with a higher risk of neonatal death, whereas for normal weight neonates, only three risk factors (1, 3, and 9) were found to be significantly associated with a higher risk for neonatal death.

Other studies in Indonesia have identified the following risk factors as being associated with a higher risk of neonatal death: low birthweight; low household income; intra-partum infection; number of previous deliveries; antenatal care provider; haemorrhage; and eclampsia (5). In our study the effect of birthweight (normal or low birthweight) did not appear as a risk factor, as it was controlled for by comparing cases and controls in the same birthweight category. Socio-economic status factors including level of income, education, poverty, accessibility, and social support were investigated in our study, but we found that only the variables of accessibility, poverty (i.e. ownership of electricity), and social support reached a statistically significant association with a higher risk of neonatal death This is also a result of our study design, as cases and controls were selected from the same village, where large variations between households in levels of income and education were unlikely.

### Complications

Complications during pregnancy or during delivery were found to be a major risk factor for neonatal death. Note that the complications were identified from the medical records of those women who delivered in a health facility or were assisted by a trained attendant and as reported by the mother where the delivery was not assisted by a trained attendant. In a previous Indonesian study, complications contributed to approximately 23.4% of neonatal deaths (10). Neonatal complications at delivery, complications during pregnancy, and having a history of complications were all found to be independently associated with neonatal deaths. With all three types of complications, the risk for neonatal death was approximately 80-fold higher compared with those who had none of these complications. The risk of having complications was higher (AOR, 7.0; 95% CI 6.6–7.4) for women with anaemia; malaria/dengue; lung, heart, and hepatic disease and there was subsequently a higher risk of neonatal death (19). However, these conditions were not identified or recorded in the medical records of all women, with no record of haemoglobin monitoring in 38% of records and malaria testing recorded for only 10% of pregnancies, although the prevalence of malaria in NTT is relatively high at 23%, the second highest prevalence after Papua (20).

### Neonatal illness and maternal knowledge of neonatal danger signs

Neonatal illness during the first month of life and knowledge of neonatal danger signs were identified as the second major risk factors for neonatal death. Although these factors have a statistically significant association, this does not necessarily point to causation, and it does not mean that more education on the danger signs of the newborn will automatically lead to a reduction in neonatal deaths. It does suggest that early detection of neonatal illness is an important step towards improving newborn survival (21), and it also suggests that aspects of the mother's previous obstetric history and her care during pregnancy impact on the risk of neonatal death. These aspects are reinforced by two other factors that emerged with significant associations and that relate to practices at birth or in early care of the newborn (early initiation of breastfeeding and use of the kangaroo method of care for low birthweight neonates). These findings also suggest the need to enhance education of mothers as part of antenatal care as well as for those discharged from health facilities after delivery (22).

### Low Apgar score

The Apgar score is an important indicator that has been associated with a higher risk of neonatal death. This score is not only useful for evaluating the clinical status of the neonate in the first minutes after birth (23), but also for determining their need for resuscitation and evaluating effectiveness (24). This study found that neonates who were born with low Apgar scores had a risk of neonatal death six times higher compared with those with a normal Apgar score. For low birthweight neonates with low Apgar scores, the risk of neonatal death was 28-fold higher compared with those with normal Apgar scores. Many other studies have found an increased risk of neonatal death for infants with low Apgar scores; for example Berglund and colleagues reported a 45-fold increased risk of neonatal death for this group (95% CI: 30–68) compared with children who had normal Apgar scores (25). Approximately 15% of neonates in this study had no recorded Apgar score, even though healthcare providers in healthcare facilities assisted the delivery. Of the controls, 8% had low Apgar scores, which is relatively high compared with other developing countries (i.e. 2.8% in Uganda) (26). Although a number of studies found poor consistency in application of the Apgar scoring, some studies indicate that a higher proportion of low Apgar scores might reflect the level of available obstetric care (24) and substandard care during delivery (27).

### Place of delivery

Place of delivery was strongly associated with a higher risk of neonatal death. Infants born at home and assisted by a TBA had a risk of death six times higher compared with infants born in a healthcare facility. Encouraging pregnant women to deliver at a health facility and not at home is challenging in NTT, as there is a cultural preference to deliver at home. Approximately 90% of respondents reported that there was a TBA in their village or in a neighbouring village, and 58% of pregnant women acknowledged that they had visited them (mainly for abdominal massage). By encouraging pregnant women to give birth in a health facility, it is estimated that the risk of neonatal mortality could be reduced by 29% (28).

### Early initiation of breastfeeding

This study reconfirmed the importance of early initiation of breastfeeding. In our study, the variable of early initiation of breastfeeding was not significant in the final model. This variable reached statistical significance only in the univariate analysis; the risk of neonatal death was 10 time higher for infants not given early initiation of breastfeeding. Failure to provide early breastfeeding has been found to increase the risk of neonatal death (29, 30). Neonates not given early breastfeeding have a risk of death more than 20 times higher compared with those breastfed early. In one study it was argued that 16% of neonatal deaths could be prevented if all infants were breastfed from Day 1 and 22% if breastfeeding started within the first hour (31).

### Strengths and limitations of this study

Most of the current evidence on risk factors associated with neonatal death in Indonesia has been derived from survey studies (4, 32). The strength of this study was its focus on a specific population of newborns, those whose death was reported to the health services, and its exploration of factors potentially amenable to health service intervention. The study sample was sufficiently large and representative to enable detection of risk factors. The study design, by reducing the influence of confounding factors outside the control of the health service, enabled a focus on areas of potential health service intervention. The sub-analyses, while necessarily using a smaller sample, did identify important differences in risk factors between key groups of newborns and suggest potential avenues for further study.

However, the study also had a number of limitations. The study population probably underrepresents births occurring outside health facilities and without the assistance of trained healthcare workers, and this group is likely to be at even higher risk for neonatal death. The data relied on medical records and maternal recall; it was thus subject to inaccuracies and missing data in medical records and to recall bias and limited health literacy on the part of the mothers. In some cases medical record information could be confirmed with mothers, but it is possible that recall bias and mothers’ interpretations of ‘health problems’ increased the reporting of this factor. However, as we were interested in identifying factors amenable to intervention, parental identification of a health problem could still serve as an important indication requiring the attention of healthcare providers.

## Conclusions and recommendations

In the design of this study, we were particularly interested in identifying risk factors that were amenable to intervention by community health services and that addressed neonatal death in normal birthweight infants and in those surviving beyond 7 days. The study confirmed a high proportion of deaths in normal birthweight infants (56%). The three risk factors associated with neonatal death in this group are amenable to community-level interventions. The high risk associated with neonatal complications at delivery suggests that parents were aware that the neonate had problems at delivery and so could potentially have sought care for these problems. However, the association with lack of knowledge of danger signs suggests that parents were not aware of the significance of the problems and/or how to seek help. The association with delayed initiation of breastfeeding may also reflect lack of knowledge, but could also reflect an ill neonate that was unable to suck. Community-level programmes to increase knowledge of danger signs and how to obtain assistance, should they occur, could address these issues.

Early neonatal death and death of low birthweight infants was associated with problems at delivery and a low Apgar score. This reinforces the importance of delivery in a health facility that have staff who have the skills and equipment needed to manage infants with birth asphyxia. This particularly applies to mothers at risk of having low birthweight infants, including mothers of young age, suffering from other complications including poor nutrition, and with premature onset of labour. Community-level services could develop interventions to arrange urgent transfer of such patients to a health facility, preferably a hospital.

A range of factors associated with higher risk of neonatal death have been identified, including risk factors for early and late neonatal death and for normal and low birthweights. Several of these risk factors could be addressed by community health service interventions, including better communication to mothers of danger signs of newborns, better access to appropriate care for newborns when parents detect problems, and more support for early initiation of breastfeeding and kangaroo care for low birthweight infants. The study reinforces the need for more focus on neonatal care during antenatal care, care immediately or shortly after delivery, and in the first month of life, as well as specific interventions to reduce neonatal deaths.

## References

[1] Lawn JE, Cousens S, Zupan J (2005). 4 million neonatal deaths: when? Where? Why?. Lancet.

[2] World Bank (2014). Mortality rate, neonatal (per 1,000 live births).

[3] Statistics Indonesia (Badan Pusat Statistik—BPS) NPaFPBB, Kementerian Kesehatan (Kemenkes—MOH), ICF International (2013). Indonesia demographic and health survey 2012.

[4] Titaley CR, Dibley MJ, Agho K, Roberts CL, Hall J (2008). Determinants of neonatal mortality in Indonesia. BMC Public Health.

[5] Djaja S, Afifah T, Sukroni A (2011). Contribution of socioeconomical and biological factor towards neonatal mortality in Indonesia. J Indonesian Med Assoc.

[6] Raharni R, Isakh BM, Diana I (2011). Profil Kematian Neonatal Berdasarkan Sosio Demografi dan Kondisi Ibu Saat Hamil di Indonesia. Buletin Penelitian Sistem Kesehatan.

[7] Arifin A, Budiasuari M, Sholikhah HH, Wasito B, Laksmiati T (2006). Kajian Pelaksanaan Kegiatan pembinaan Kesehatan Reproduksi untuk Mempercepat Penurunan AKI dan AKB.

[8] Dinas Kesehatan Provinsi NTT (2009). Pedoman Revolusi KIA di NTT: Percepatan Penurunan Kematian Ibu dan Bayi Baru Lahir (Semua Persalinan Dilaksanakan di Fasilitas Kesehatan yang Memadai).

[9] Coffey International – University of Melbourne and AusAID (2014). Australia-Indonesia partnership for maternal neonatal health: 10th progress report.

[10] Astuti WD, Sholikhah HH, Angkasawati TJ (2010). Estimasi Risiko Penyebab Kematian Neonatal di Indonesia Tahun 2007. Buletin Penelitian Sistem Kesehatan.

[11] Malqvist M (2011). Neonatal mortality: an invisible and marginalised trauma. Glob Health Action.

[12] Singh A, Kumar A, Kumar A (2013). Determinants of neonatal mortality in rural India, 2007–2008. Peer J.

[13] Katz J, West KP, Khatry SK, Christian P, LeClerq SC, Pradhan EK (2003). Risk factors for early infant mortality in Sarlahi district, Nepal. Bull World Health Organ.

[14] Bjerregaard-Andersen M, Lund N, Jepsen FS, Camala L, Gomes MA, Christensen K (2012). A prospective study of twinning and perinatal mortality in urban Guinea-Bissau. BMC Pregnancy Childbirth.

[15] Vogel JP, Torloni MR, Seuc A, Betran AP, Widmer M, Souza JP (2013). Maternal and perinatal outcomes of twin pregnancy in 23 low- and middle-income Countries. PLoS One.

[16] Hosmer DW, Lemeshow S (2000). Applied logistic regression.

[17] Kleinbaum D, Klein M (2002). Logistic regression.

[18] StataCorp LP (2009). Stata base reference manual, release 11.

[19] Lumbiganon P, Laopaiboon M, Intarut N, Vogel JP, Souza JP, Gülmezoglu AM (2014). Indirect causes of severe adverse maternal outcomes: a secondary analysis of the WHO Multicountry Survey on Maternal and Newborn Health. BJOG.

[20] Ministry of Health (2013). Basic health research – RISKESDA 2013.

[21] Choi Y, El Arifeen S, Mannan I, Rahman SM, Bari S, Darmstadt GL (2010). Can mothers recognize neonatal illness correctly? Comparison of maternal report and assessment by community health workers in rural Bangladesh. Trop Med Int Health.

[22] Sandberg J, Odberg Pettersson K, Asp G, Kabakyenga J, Agardh A (2014). Inadequate knowledge of neonatal danger signs among recently delivered women in southwestern rural Uganda: a community survey. PLoS One.

[23] Portman RJ, Carter BS, Gaylord MS, Murphy MG, Thieme RE, Merenstein GB (1990). Predicting neonatal morbidity after perinatal asphyxia: a scoring system. Am J Obstet Gynecol.

[24] Ehrenstein V (2009). Association of Apgar scores with death and neurologic disability. Clin Epidemiol.

[25] Moster D, Lie RT, Irgens LM, Bjerkedal T, Markestad T (2001). The association of Apgar score with subsequent death and cerebral palsy: a population-based study in term infants. J Pediatr.

[26] Ondoa-Onama C, Tumwine JK (2003). Immediate outcome of babies with low Apgar score in Mulago Hospital, Uganda. East Afr Med J.

[27] Berglund S, Pettersson H, Cnattingius S, Grunewald C (2010). How often is a low Apgar score the result of substandard care during labour?. BJOG.

[28] Tura G, Fantahun M, Worku A (2013). The effect of health facility delivery on neonatal mortality: systematic review and meta-analysis. BMC Pregnancy Childbirth.

[29] Debes AK, Kohli A, Walker N, Edmond K, Mullany LC (2013). Time to initiation of breastfeeding and neonatal mortality and morbidity: a systematic review. BMC Public Health.

[30] Mullany LC, Katz J, Li YM, Khatry SK, LeClerq SC, Darmstadt GL (2008). Breast-feeding patterns, time to initiation, and mortality risk among newborns in Southern Nepal 1, 2. J Nutr.

[31] Edmond KM, Zandoh C, Quigley MA, Amenga-Etego S, Owusu-Agyei S, Kirkwood BR (2006). Delayed breastfeeding initiation increases risk of neonatal mortality. Pediatrics.

[32] Hatt L, Stanton C, Ronsmans C, Makowiecka K, Adisasmita A (2009). Did professional attendance at home births improve early neonatal survival in Indonesia?. Health Policy Plan.

